# Effects of *Moringa oleifera* supplementation on immune and nutritional biomarkers in adults living with HIV: a systematic review and meta-analysis

**DOI:** 10.3389/fnut.2025.1667158

**Published:** 2025-09-08

**Authors:** Dachuan Jin, Shunqin Jin, Tao Zhou, Guoping Sheng, Peng Gao, Guangming Li

**Affiliations:** ^1^Translational Medicine Research Center, Zhengzhou Sixth People's Hospital, Zhengzhou, China; ^2^Department of Radiology, Hebei Medical University, Shijiazhuang, China; ^3^Department of Geriatric Medicine, Key Laboratory of Cardiovascular Proteomics of Shandong University, Qilu Hospital of Shandong University, Jinan, China; ^4^Key Laboratory of Artificial Organs and Computational Medicine of Zhejiang Province, Shulan (Hangzhou) International Medical College, Zhejiang Shuren University, Hangzhou, China; ^5^Department of Liver Disease, Zhengzhou Sixth People's Hospital, Zhengzhou, China

**Keywords:** *Moringa oleifera*, HIV, immune function, BMI, meta-analysis

## Abstract

**Background:**

*Moringa oleifera* (*MO*) is widely used as an adjunctive therapy for individuals living with HIV (PLWH) due to its nutritional and immune-modulating properties.

**Objective:**

To systematically evaluate the effects of MO supplementation on immune and nutritional indicators in Human Immunodeficiency Virus (HIV)-infected adults.

**Methods:**

We conducted a systematic review and meta-analysis by searching PubMed, EmBase, Web of Science, and Cochrane Library to include studies assessing the impact of MO supplementation on immune and nutritional markers, such as CD4^+^ T cell count, BMI, white blood cell (WBC) count, and platelet (PLT) count in PLWH. Data were pooled using random-effects or fixed-effects models, and subgroup and meta-regression analyses were performed to assess sources of heterogeneity.

**Results:**

A total of seven articles (eight study datasets) were included. MO supplementation significantly increased CD4^+^ T cell count [standardized mean differences (SMD) = 1.4, 95% CI 0.59–2.20, *p* < 0.001], WBC (SMD = 0.22, 95% CI 0.02–0.42, *p* = 0.030), and PLT count (SMD = 3.14, 95% CI 2.37–3.92, *p* < 0.001), with a significant improvement in BMI (SMD = 0.29, 95% CI 0.03–0.55, *p* = 0.028). Subgroup analysis demonstrated consistent effects in both randomized controlled trials (RCTs) and non-RCTs, while meta-regression indicated that dosage influences outcomes (*p* = 0.007). Further studies with larger sample sizes are warranted.

**Conclusions:**

*MO* supplementation significantly improves immune function and nutritional status in PLWH. Further high-quality studies are needed to confirm its efficacy and safety.

**Systematic review registration:**

https://www.crd.york.ac.uk/prospero/display_record.php?ID=CRD420251000927, PROSPERO: CRD420251000927.

## 1 Introduction

Human Immunodeficiency Virus (HIV) remains a significant global public health challenge. According to the latest data from the Joint United Nations Programme on HIV/AIDS (UNAIDS), ~38 million people are currently living with HIV worldwide in 2023, and around 650,000 deaths occur annually due to HIV-related complications (1–3). While the virus is prevalent in high-income countries to some extent, its incidence and mortality rates are significantly higher in low- and middle-income countries, where limited access to timely diagnosis, antiretroviral therapy (ART), and supportive care exacerbates disease progression (2, 3). This disproportionate burden underscores the importance of understanding the virus's pathogenesis—HIV primarily targets CD4^+^ T lymphocytes, progressively impairing immune function and rendering individuals vulnerable to opportunistic infections and malignancies (4, 5). Despite substantial progress in antiretroviral therapy (ART) over recent decades—which has notably extended life expectancy and improved quality of life for many patients—current treatment strategies still face several limitations. Challenges such as drug resistance, adverse drug effects, and insufficient access to medical resources in certain regions hinder the consistent and effective delivery of ART (6). Moreover, although ART can effectively suppress viral replication, many patients continue to exhibit immunological abnormalities, including low CD4^+^ T cell counts, leukopenia, and thrombocytopenia (7–9). These issues reflect the direct damage inflicted by the virus on the immune system and indicate a persistent state of immunosuppression (10–12). Furthermore, HIV-infected individuals frequently suffer from malnutrition, exacerbated by the chronic nature of the disease and long-term drug toxicity. Low body mass index (BMI) is common in this population, adversely affecting overall health and increasing the risk of comorbid conditions (13–15). Collectively, these observations indicate that relying solely on existing ART regimens is insufficient to achieve comprehensive improvements in immune restoration and nutritional status among HIV-infected individuals. There is an urgent need to develop and explore novel adjunctive therapeutic strategies.

*Moringa oleifera* (MO), also known as “*moringa*” or “*quiabo-de-quina*,” is widely cultivated in the Middle East, Africa, and Asia (16, 17). In recent years, *Moringa oleifero* has garnered increasing attention in the medical community due to its rich content of essential nutrients and bioactive compounds (18, 19). A growing body of research has explored its potential role in enhancing immune function and improving nutritional status, particularly among individuals living with HIV (PLWH). Evidence suggests that *Moringa oleifera* may positively influence key immunological markers—such as CD4^+^ T cells, white blood cells (WBC), and platelets (PLT)—as well as BMI (18, 20–23). However, the findings remain inconsistent, and comprehensive synthesis and quantitative evaluation of the available data are lacking. For instance, Ogbuagu et al. (18) reported that *Moringa oleifera* significantly increased CD4^+^ T cell count in HIV-infected individuals, potentially enhancing treatment outcomes. In contrast, Tshingani et al. did not observe a significant increase in CD4^+^ T cell counts following *Moringa* supplementation (24).

This study aims to conduct a systematic review and meta-analysis to comprehensively evaluate the effects of *Moringa oleifera* on CD4^+^ T cell count, WBC count, PLT count, and BMI in adults living with HIV. It will thereby provide more robust evidence to support clinical decision-making regarding adjunctive therapeutic strategies.

## 2 Methods

We conducted this systematic review and meta-analysis in accordance with the guidelines outlined in the Preferred Reporting Items for Systematic Reviews and Meta-Analyses (PRISMA) statement (25) and officially registered the protocol for this study with PROSPERO under the registration code CRD420251000927.

### 2.1 Search strategy

A comprehensive literature search was performed across multiple electronic databases, including PubMed, EmBase, Web of Science, and Cochrane Library, covering studies published up to March 30th, 2025. The search terms incorporated Medical Subject Headings (MeSH) and free-text keywords such as “*Moringa oleifera*,” “*Drumsticktree,” “HIV,” “HTLV-III,” “Human Immunodeficiency Virus,” “Human T-Cell Lymphotropic Virus Type III,” “AIDS,”* and “*Acquired Immune Deficiency Syndrome*.” Boolean operators (AND, OR) were used to refine the search. As an illustration, the complete search algorithm used for PubMed is provided in Table 1. No restrictions were placed on language or geographical location. In addition, the reference lists of included articles and relevant reviews were manually examined to identify any additional eligible studies.

**Table 1:** Search strategies on PubMed.

#1	*Moringa oleifera*[MeSH Terms]
#2	*Moringa oleifera*[Title/Abstract] OR *Moringa oleifera*s[Title/Abstract] OR *oleifera, Moringa*[Title/Abstract] OR Drumsticktree[Title/Abstract] OR Drumsticktrees[Title/Abstract] OR *Moringa*[Title/Abstract] OR *Moringa*s[Title/Abstract]
#3	#1 OR #2
#4	hiv[MeSH Terms]
#5	HIV[Title/Abstract] OR HTLV-III[Title/Abstract] OR Human Immunodeficiency Virus[Title/Abstract] OR Immunodeficiency Virus, Human[Title/Abstract] OR Immunodeficiency Viruses, Human[Title/Abstract] OR Virus, Human Immunodeficiency[Title/Abstract] OR Viruses, Human Immunodeficiency[Title/Abstract] OR Human Immunodeficiency Viruses[Title/Abstract] OR Human T Cell Lymphotropic Virus Type III[Title/Abstract] OR Human T-Cell Lymphotropic Virus Type III[Title/Abstract] OR Human T-Cell Leukemia Virus Type III[Title/Abstract] OR Human T Cell Leukemia Virus Type III[Title/Abstract] OR LAV-HTLV-III[Title/Abstract] OR Lymphadenopathy-Associated Virus[Title/Abstract] OR Lymphadenopathy Associated Virus[Title/Abstract] OR Lymphadenopathy-Associated Viruses[Title/Abstract] OR Viruses, Lymphadenopathy-Associated[Title/Abstract] OR Virus, Lymphadenopathy-Associated[Title/Abstract] OR Human T Lymphotropic Virus Type III[Title/Abstract] OR Human T-Lymphotropic Virus Type III[Title/Abstract] OR AIDS Virus[Title/Abstract] OR AIDS Viruses[Title/Abstract] OR Virus, AIDS[Title/Abstract] OR Viruses, AIDS[Title/Abstract] OR Acquired Immune Deficiency Syndrome Virus[Title/Abstract] OR Acquired Immunodeficiency Syndrome Virus[Title/Abstract] OR Acquired Immunodeficiency Syndrome[Title/Abstract] OR AIDS[Title/Abstract] OR Immunodeficiency Syndrome, Acquired[Title/Abstract] OR Acquired Immunodeficiency Syndromes[Title/Abstract] OR Immunodeficiency Syndromes, Acquired[Title/Abstract] OR Syndrome, Acquired Immunodeficiency[Title/Abstract] OR Syndromes, Acquired Immunodeficiency[Title/Abstract] OR Acquired Immune Deficiency Syndrome[Title/Abstract] OR Acquired Immuno-Deficiency Syndrome[Title/Abstract] OR Acquired Immuno Deficiency Syndrome[Title/Abstract] OR Acquired Immuno-Deficiency Syndromes[Title/Abstract] OR Immuno-Deficiency Syndrome, Acquired[Title/Abstract] OR Immuno-Deficiency Syndromes, Acquired[Title/Abstract] OR Syndrome, Acquired Immuno-Deficiency[Title/Abstract] OR Syndromes, Acquired Immuno-Deficiency[Title/Abstract] OR Immunologic Deficiency Syndrome, Acquired[Title/Abstract]
#6	#4 OR #5
#7	#3 AND #6

### 2.2 Eligibility criteria

Studies were selected based on predefined Population, Intervention, Comparison, Outcome, and Study design (PICOS) criteria (26): (1) population: adults aged 18 years or older diagnosed with HIV. (2) Intervention: eligible interventions included any oral form of *Moringa oleifera* supplementation, regardless of preparation type or dosage. Co-interventions (e.g., ART, nutritional counseling) were permitted if applied equally to both the intervention and control groups. Studies using multi-herbal formulations were included if the specific effects of *Moringa oleifera* could be isolated or were clearly dominant in the formulation. (3) Comparison: placebo, no supplementation, or standard treatment. (4) Outcomes: primary outcomes included markers of immune function (e.g., CD4^+^ T cell count, WBC, and PLT). Secondary outcomes involved BMI. (5) Study design: interventional studies evaluating the effects of *Moringa oleifera* supplementation in HIV-positive adults were included. Eligible studies comprised randomized controlled trials (RCTs) and non-randomized interventional studies (e.g., single-arm pre-post trials). Studies were required to report outcome data before and after the intervention.

### 2.3 Exclusion criteria

Studies were excluded if they met any of the following conditions: (1) multi-herbal formulations without isolated *Moringa* effects: studies where the specific effects of *Moringa oleifera* could not be isolated, or it was not the dominant ingredient. (2) Non-supplementation use: *Moringa oleifera* used solely for non-supplementation purposes (e.g., topical, cosmetic, or external applications). (3) Unequal co-interventions: co-interventions (e.g., ART, nutritional counseling) applied unequally between intervention and control groups. (4) Non-clinical studies: *in vitro* or animal studies. (5) Non-interventional designs: observational studies, case reports, cohort studies, or pre-post designs without a defined intervention. (6) Lack of relevant outcome data: no report of immune function markers (e.g., CD4+ T cell count, WBC, PLT) or BMI. (7) Inadequate intervention duration: intervention period < 4 weeks, unless strong evidence suggests that shorter-term effects are highly relevant to the research question.

### 2.4 Data extraction and management

Two independent reviewers (S.J. and T.Z) conducted the selection process and extracted relevant data using a standardized form. Disagreements were resolved through discussion or consultation with a third reviewer. Extracted information comprised: authorship, year of publication, nation or region, sample size, duration of intervention, study design, characteristics of participants (e.g., mean age), and reported outcomes of interest. Where necessary, corresponding authors were contacted to obtain missing data.

### 2.5 Methodological quality and bias assessment

To assess the methodological quality and risk of bias of the included studies, we applied different tools according to the study design (27). For randomized controlled trials (RCTs), we used the Cochrane Collaboration's Risk of Bias 2 (RoB 2) tool (https://methods.cochrane.org/risk-bias-2), which evaluates five domains: bias arising from the randomization process, bias due to deviations from intended intervention, bias due to missing outcome data, bias in measurement of the outcome, and bias in selection of the reported results. Judgments for each domain and overall risk of bias were categorized as “low risk,” “some concerns,” or “high risk” (28). Two reviewers (S.J. and T.Z.) independently performed the assessments, and any disagreements were resolved through discussion and consensus.

For non-randomized studies of interventions, we applied the Risk of Bias in Non-randomized Studies-of Interventions (ROBINS-1) tool (29). This tool examines the risk of bias across seven key domains: bias due to confounding, bias due to selection of participants, bias due to deviations from intended interventions, bias due to missing data, bias in measurement of outcomes, bias in selection of the reported result, and overall risk of bias. Judgments for each domain and overall risk of bias were categorized as low, moderate, serious, or critical. An “unclear” rating was used when information was insufficient to permit a clear judgment.

Assessment of publication bias was planned according to the number of included studies per outcome. Specifically, if ≥10 studies were available, funnel plot analysis and statistical tests (Begg's rank correlation and Egger's regression asymmetry) would be performed. If fewer than 10 studies were available, no publication bias assessment would be conducted, in line with the Cochrane Handbook recommendations to avoid unreliable interpretation due to low statistical power.

### 2.6 Statistical analysis

Meta-analyses (24) were conducted using STATA software (version 15.1, StataCorp, College Station, TX). For continuous outcomes, standardized mean differences (SMDs) with 95% confidence intervals (CIs) were calculated. Heterogeneity across studies was assessed using the *I*^2^ statistic and Cochran's *Q* test, with *I*^2^ values above 50% indicating substantial heterogeneity, for which random-effects models were applied. In contrast, fixed-effect models were used for outcomes with low heterogeneity (*I*^2^ < 50%). To further explore potential sources of heterogeneity, subgroup analyses were planned according to predefined study-level factors such as intervention dose, duration, or participant characteristics. In addition, meta-regression analyses were applied to examine the relationship between effect sizes and selected covariates. A two-tailed *p*-value < 0.05 was considered statistically significant.

## 3 Results

### 3.1 Literature search results

**Figure 1:**
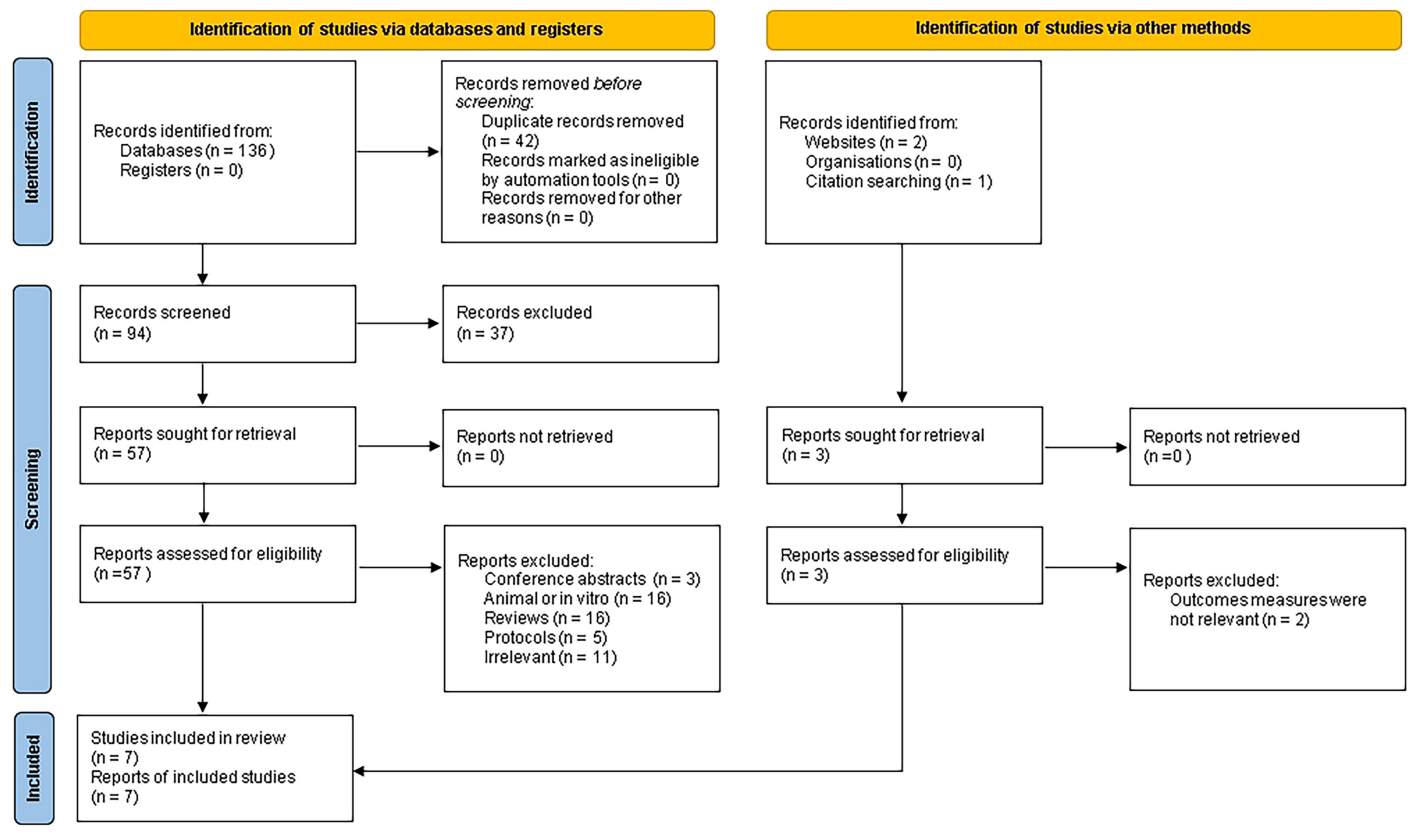
PRISMA flowchart.

The literature selection process was illustrated in Figure 1. A total of 136 articles were initially identified from four electronic databases, and an additional three relevant articles were obtained from other sources. After removing 42 duplicates, 97 articles remained. Based on titles and abstracts, 37 articles that did not meet the inclusion criteria were excluded. After a full-text review, 53 articles were excluded. Ultimately, seven articles were included in the final meta-analysis (7, 18, 20, 24, 30–33).

### 3.2 Study characteristics

This study included seven articles, comprising eight study datasets (1,022 participants). The datasets consisted of six randomized controlled trials and two prospective pre-post datasets reported within the same conference abstract.

A total of seven articles, comprising eight study datasets and involving 1,022 participants (520 patients and 502 controls), were included in this study. The datasets consisted of six randomized controlled trials (20, 30–33) and two prospective pre-post studies, both of which were reported within the same conference abstract by Ogbuagu et al. (18) and treated as two independent datasets in our analysis (Ogbuagu 2016a and Ogbuagu 2016b). All included studies were conducted in Africa, with five from Nigeria (18, 20, 30, 33), and each from Uganda (31), Kenya (32), and the Democratic Republic of the Congo (24). *Moringa oleifera* was widely used as a traditional herbal remedy in many African countries for enhancing immunity, improving nutritional status, and managing HIV. The publication years of the included studies ranged from 2016 (18) to 2024 (31, 32). The smallest study involved 30 participants, while the largest included 208. The daily dose of *Moringa oleifera* administered ranged from 0.2 to 20 grams, with intervention durations varying from 2 to 12 months. Detailed characteristics of all included studies were presented in Table 2.

**Table 2:** Characteristics of included studies.

**Study**	**Nation**	***N***	**Age (mean±SD)**	**Intervention**	**Study design**	**Outcomes of interest**
Ogbuagu 2016a (18)^*^	Nigeria	15	NA	T: MO 20g/d × 2 m	Single-arm pre-post study	CD4 count
		15	NA	C: No Intervention		
Ogbuagu 2016b (18)^*^	Nigeria	25	NA	T: MO 20 g/d × 2 m	Single-arm pre-post study	CD4 count
		25	NA	C: No Intervention		
Gambo 2022a (30)	Nigeria	89	NA	T: 5 g × 3 times/day × 6 m	RCT	CD4 count
		88	NA	C: Placebo		
Gambo 2022b (33)	Nigeria	89	NA	T: 5 g × 3 times/day × 6 m	RCT	BMI
		88	NA	C: Placebo		
Twinomujuni 2024 (31)	Uganda	86	39.2 ± 9.90	T: Artemisia(4 g/d) + MO(10 g/d) × 12 m	RCT	CD4 count, WBC, platelet
		88	40.9 ± 9.80	C: Artemisia(4 g/d) × 12 m		
Tshingan 2017 (24)	Congo	29	49.5 ± 10.75	T: MO 30 g/d × 6 m	RCT	CD4 count, BMI
		29	47.0 ± 10.75	C: Placebo		
Aprioku 2022 (20)	Nigeria	104	NA	T: HAART+ MO 0.2 g/d × 3 m	RCT	CD4 count, WBC, platelet
		104	NA	C: HAART		
Wilbroda 2024 (32)	Kenya	83	NA	T: MO 10 g/d × 6 m	RCT	CD4 count
		65	NA	C: Placebo		

MO, Moringa oleifera; T, treatment; C, control; g, gram; d, day; m, month; RCT, randomized controlled trial; BMI, body mass index; WBC, white blood cell; HAART, highly active antiretroviral therapy.

^*^Data for both Ogbuagu 2016a and Ogbuagu 2016b were extracted from the same conference abstract (18), which reported two separate intervention groups.

### 3.3 Effects on immune parameters and BMI

#### 3.3.1 Immune parameters

**Figure 2:**
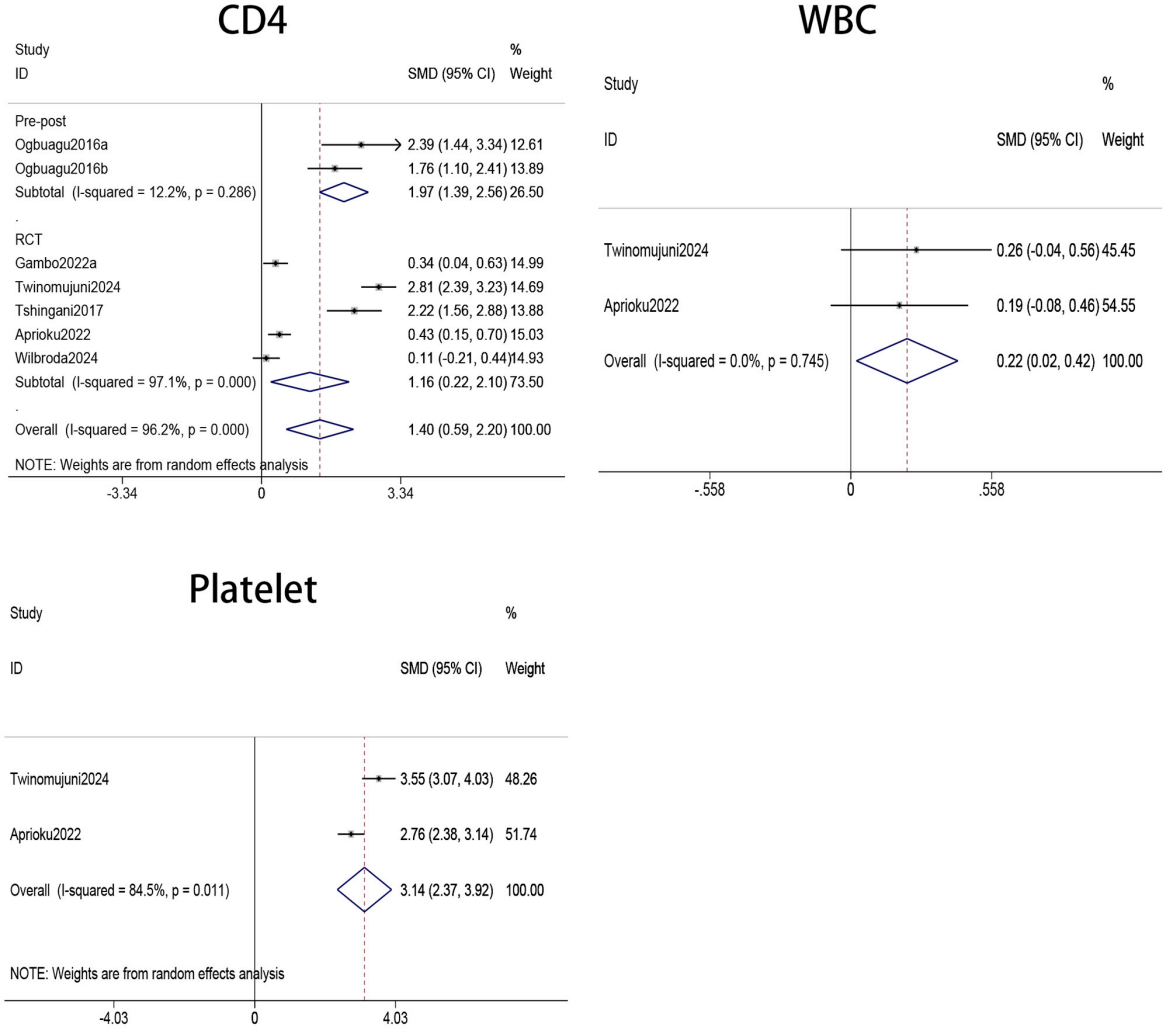
Forest plots of CD4, WBC, and PLT counts in PLWH. WBC, white blood cell; PLT, platelet; PLWH, people living with HIV.

The immune parameters evaluated in our study included CD4^+^ T cell count, WBC count, and platelet count. Figure 2 presents the pooled analysis results of CD4^+^ T cell counts for seven treatment groups (18, 20, 30–32). The findings indicated that CD4^+^ T cell counts were significantly higher in patients receiving *Moringa oleifera* supplementation than controls (SMD 1.4, 95% CI 0.59–2.20, *p* < 0.001). Heterogeneity analysis revealed a high level of heterogeneity (*I*^2^ = 96.2%, *p* < 0.001); therefore, a random-effects model was applied for this outcome.

The pooled analysis of two treatment groups (20, 31) showed that WBC counts were significantly higher in HIV patients receiving *Moringa oleifera* supplementation compared to those in the control group without supplementation (SMD 0.22, 95% CI 0.02–0.42, *p* = 0.030). Heterogeneity analysis indicated a low level of heterogeneity (*I*^2^ = 0.0%, *p* = 0.745); therefore, a fixed-effects model was used to analyze the effect on WBC count.

Platelets were involved in innate and adaptive immunity, as they could recognize viruses and release mediators such as IL-1β and CD40L, thereby influencing immune responses. Consequently, platelet count and function are closely associated with the state of the immune system. Our meta-analysis of two studies (20, 31) involving platelets showed that *Moringa oleifera* supplementation significantly increased platelet levels in HIV patients (SMD = 3.14, 95% CI 2.37–3.92, *p* < 0.001). Heterogeneity analysis indicated a high level of heterogeneity (*I*^2^ = 84.5%, *p* = 0.011); therefore, a random-effects model was applied to analyze the effect on platelet count.

#### 3.3.2 BMI

**Figure 3:**
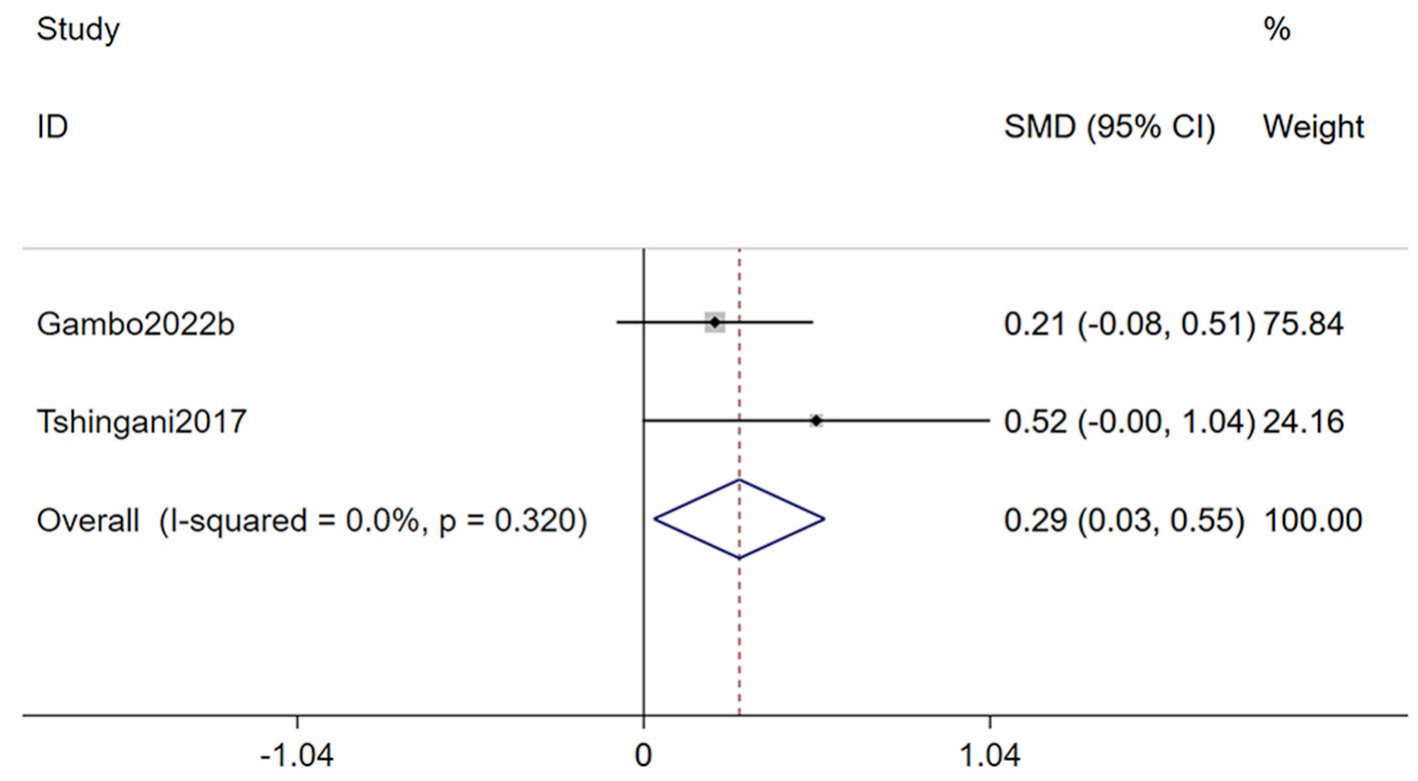
Forest plot of BMI in PLWH. BMI, body mass index; PLWH, people living with HIV.

Two studies (24, 33) reporting on body mass index, were included in this analysis (Figure 3). The meta-analysis demonstrated that *Moringa oleifera* supplementation significantly improved BMI in HIV patients (SMD 0.29, 95% CI 0.03–0.55, *p* = 0.028). Heterogeneity analysis indicated a low level of heterogeneity (*I*^2^ = 0.0%, *p* = 0.320); therefore, a fixed-effects model was used for this outcome.

### 3.4 Subgroup and meta-regression analysis

**Figure 4:**
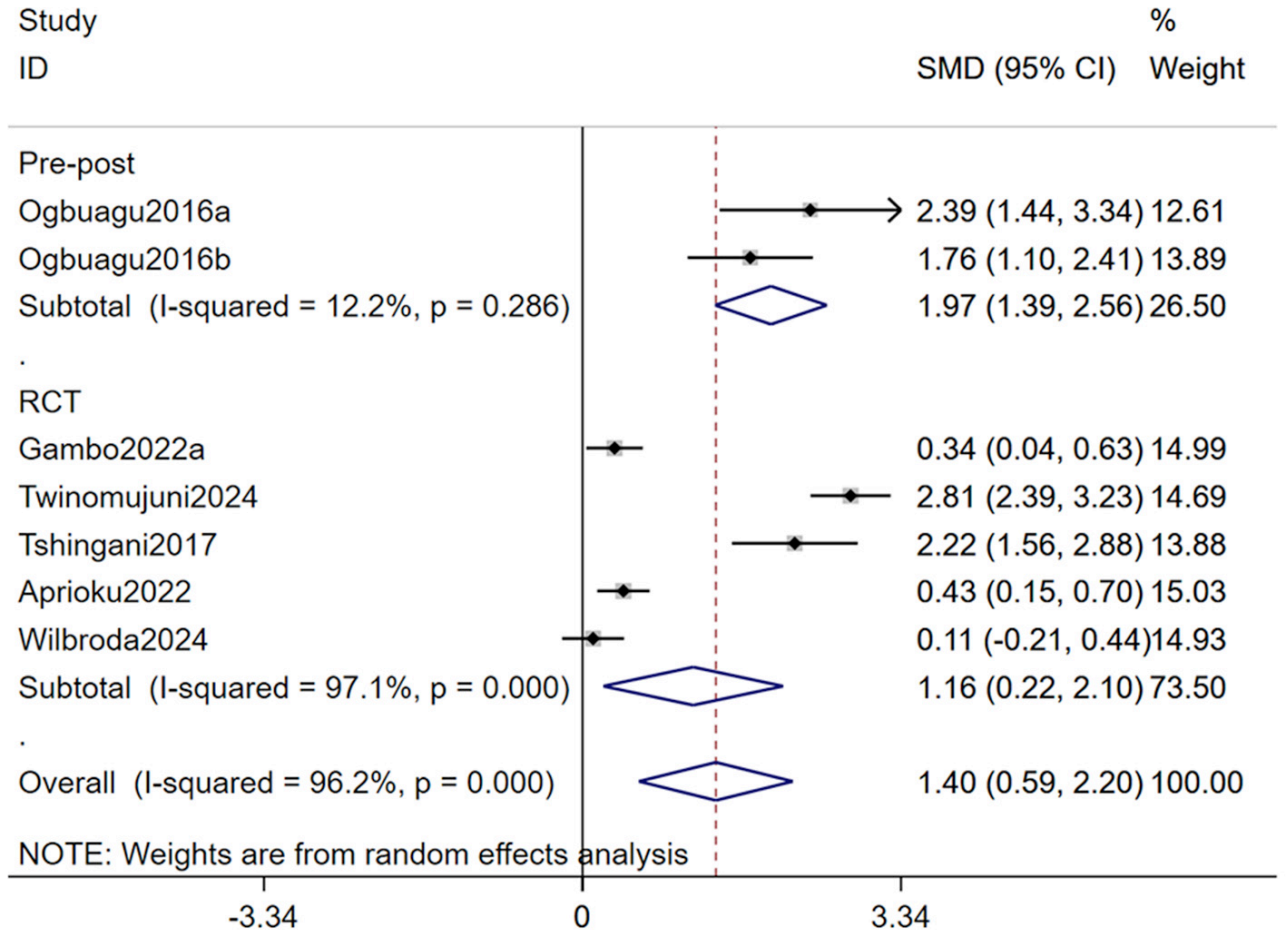
Forest plot for subgroup analysis by study design for CD4+ T cell count in PLWH. PLWH, people living with HIV.

As both subgroup analysis and meta-regression require a minimum number of included studies, we conducted a subgroup analysis of CD4^+^ T cell count based on study design and a meta-regression analysis based on dosage and duration of *Moringa oleifera* supplementation. Our findings indicated that the subgroup analysis partially reduced heterogeneity, suggesting that variations in study design influenced the outcomes. Nevertheless, *Moringa oleifera* supplementation significantly increased CD4^+^ T cell counts in both RCTs (SMD: 1.16, 95% CI 0.22–2.10, *p* = 0.001) and non-RCTs (SMD: 1.97, 95% CI: 1.39–2.56, *p* < 0.001; Figure 4). Meta-regression analysis revealed that dosage had a significant impact on treatment outcomes (*p* = 0.007), whereas treatment duration did not show a statistically significant effect (*p* = 0.260; Table 3).

**Table 3:** Meta-regression of CD4 count by dosage and treatment duration.

**Variables**	**Coef**.	**Std. Err**.	***z***	***P*>|*z*|**	**95% conf. interval**	
dose	−0.0155011	0.0145248	−1.07	0.286	−0.0439691	0.0129669
_cons	0.6048760	0.2253493	2.68	0.007	0.1631995	1.0465530
duration	0.0098455	0.0057784	1.70	0.088	−0.0014799	0.0211709
_cons	0.1952459	0.1734611	1.13	0.260	−0.1447317	0.5352234

### 3.5 Risk of bias (RoB) analysis

**Figure 5:**
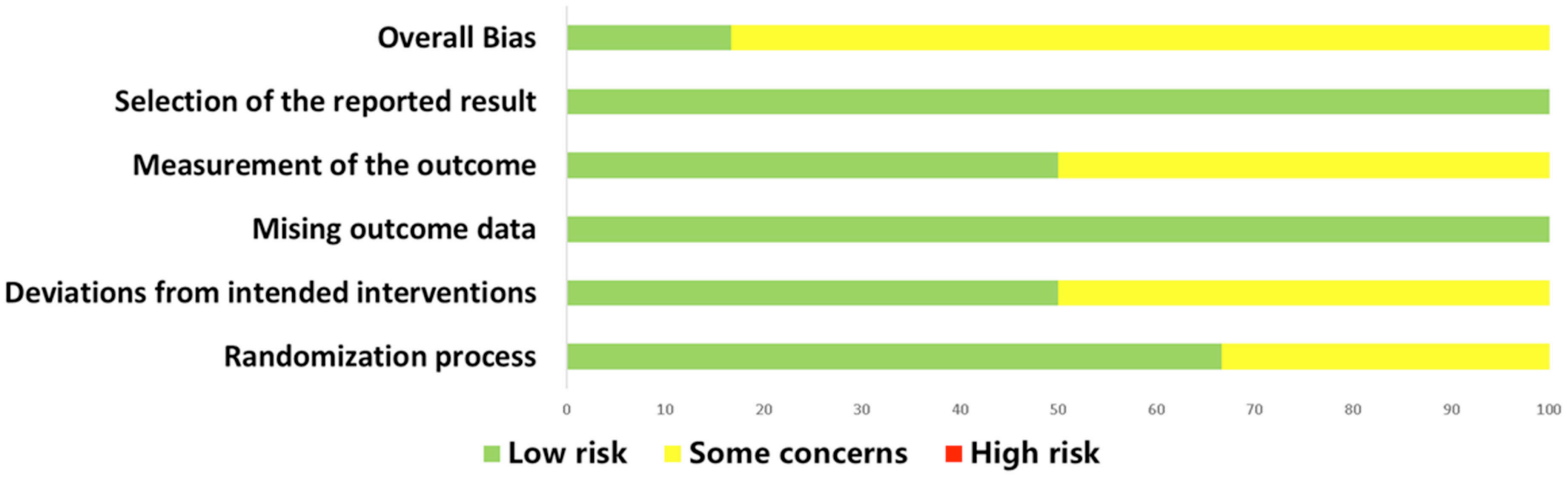
Summary of risk of bias judgments for all included RCTs using the revised Cochrane RoB 2 tool. RCT, randomized controlled trial.

**Figure 6:**
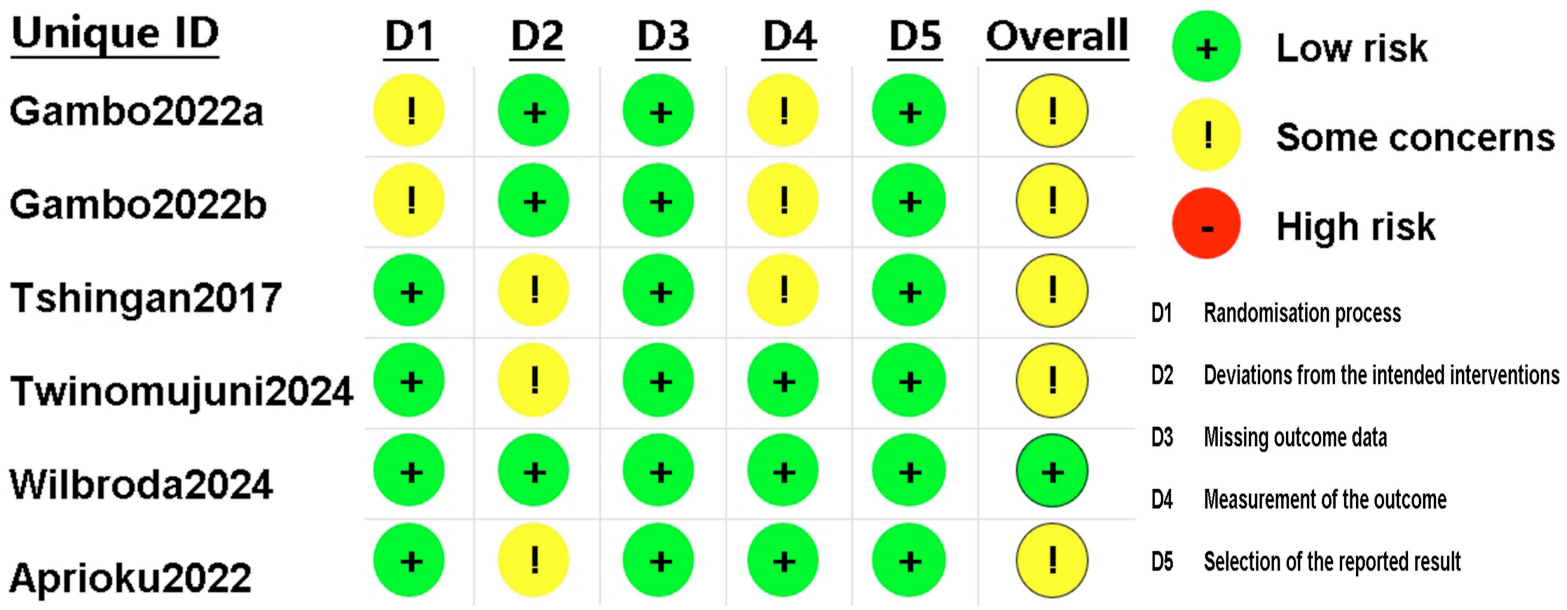
Risk of bias assessment for included RCTs using the revised Cochrane RoB 2 tool. RCT, randomized controlled trial.

The assessment results for the RCT studies are presented in Figures 5, 6. Using the latest Cochrane Risk of Bias 2 (RoB 2) tool, most trials were judged to be at low risk of bias in the majority of domains. In Domain 1 (bias arising from the randomization process), two studies were rated as having *Some concerns* because they reported randomization without sufficient detail on sequence generation and did not describe an allocation concealment mechanism (30, 33). In Domain 2 (bias due to deviations from intended interventions), three studies were judged as having *Some concerns* owing to unclear or incomplete reporting of blinding of participants and/or personnel, with no explicit statement on the use of an intention-to-treat approach (20, 24, 31) In Domain 4 (bias in measurement of the outcome), three studies were rated as having *Some concerns* because the blinding status of outcome assessors was not reported, leaving the possibility of measurement bias (24, 30, 33). In total, five studies were rated as having *Some concerns* in at least one domain (20, 24, 30, 31, 33), while one study was rated as low risk of bias across all domains (32).

**Figure 7:**
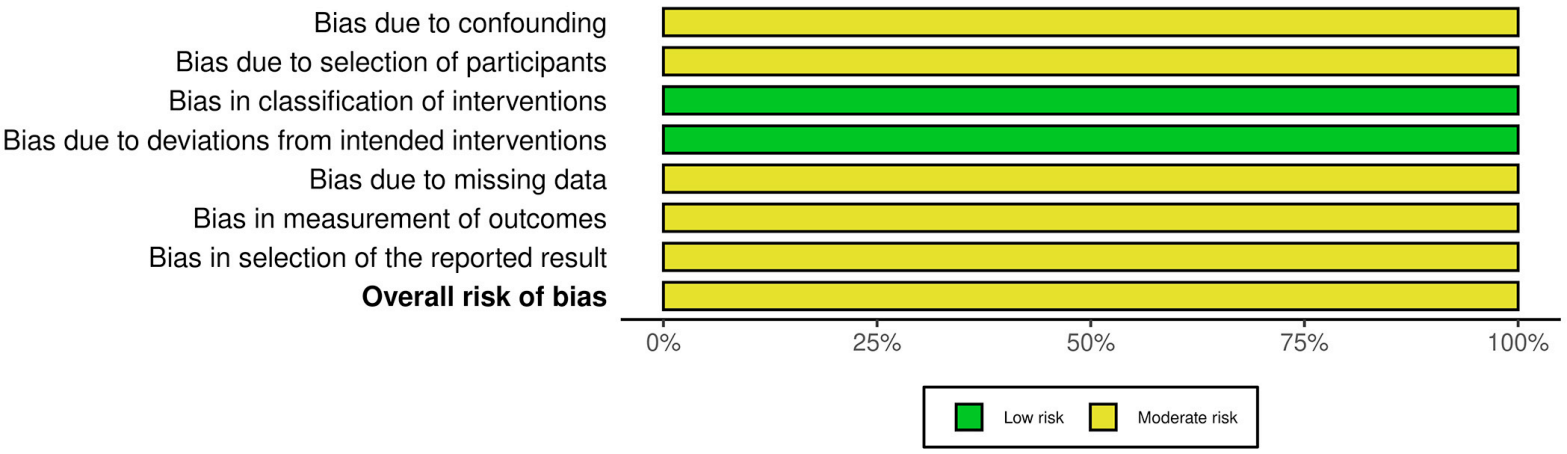
Risk of bias assessment of non-randomized studies of intervention using ROBINS-1 Tool.

**Figure 8:**
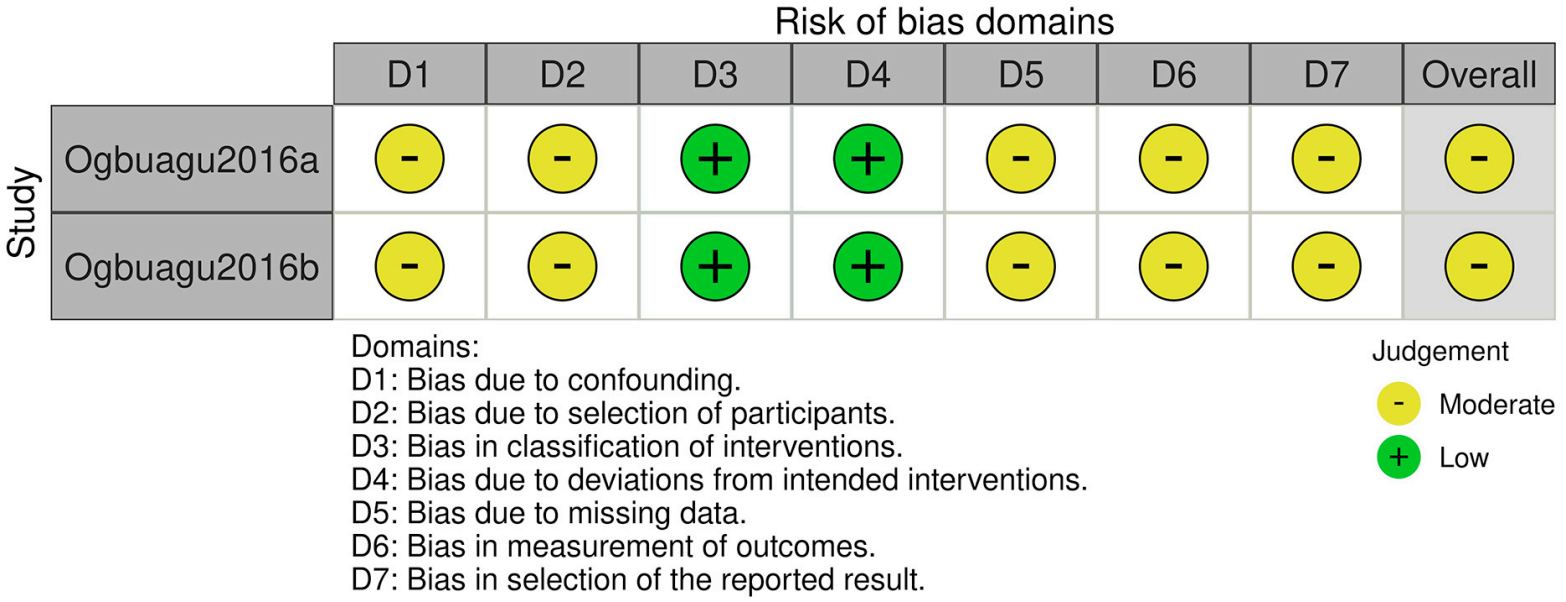
Risk of bias assessment using ROBINS-I tool for individual non-randomized studies of intervention.

The two included non-RCT studies originated from the same publication (18). We assessed the risk of bias using the ROBINS-I tool, with the results presented in Figures 7, 8. Both studies were judged to have a *moderate risk of bias*. Specifically, they were rated as *low risk* in the domains of bias in classification of interventions and bias due to deviations from intended interventions. However, *some concerns* were identified in the remaining five domains: bias due to confounding (no adjustment for key prognostic variables such as baseline immune status or nutritional intake), bias due to selection of participants (unclear recruitment procedures and potential for non-consecutive inclusion), bias due to missing data (incomplete reporting of follow-up or attrition rates), bias in measurement of outcomes (no information on blinding of outcome assessors), and bias in selection of the reported result (lack of a pre-specified analysis plan, raising the possibility of selective reporting). These limitations prevented a “low risk” judgment, although no domain was rated as “serious” or “critical” risk of bias.

### 3.6 Publication bias assessment

No publication bias assessment was conducted because none of the outcomes included 10 or more studies, which was the prespecified threshold for performing funnel plot analysis and statistical tests (Begg's rank correlation and Egger's regression asymmetry). This decision followed the recommendations of the Cochrane Handbook to avoid unreliable interpretation due to low statistical power when fewer studies are available.

### 3.7 Adverse events

Among the seven included articles, two explicitly assessed the safety of *Moringa oleifera*, and neither reported any significant adverse events (31, 32). The study by Wilbroda et al. (32) found that *Moringa oleifera* had no detrimental effects on renal function, with glomerular filtration rates remaining within the normal range in both the intervention and control groups. They concluded that *Moringa oleifera* did not significantly affect renal function and considered it non-toxic. Similarly, Twinomujun et al. (31) did not observe any adverse events of *Moringa oleifera* on liver and kidney functions, nor were any subjective adverse events reported by participants. These findings suggested that *Moringa oleifera* was well-tolerated and safe within the commonly used dosage range. Nevertheless, further research is needed to evaluate the safety of long-term and high-dose use of *Moringa oleifera*, particularly in immunocompromised populations such as individuals living with HIV, to ensure its clinical safety and scientific validity.

## 4 Discussion

By synthesizing evidence from seven publications involving eight studies with a total of 1,022 participants, this study is the first to employ a meta-analytic approach to evaluate the effects of *Moringa oleifera* supplementation on immune function and BMI in adults with HIV (18, 20, 24, 30–33). The findings indicate that *Moringa oleifera* supplementation significantly improves CD4^+^ T cell, WBC, and platelet counts. In addition, it has a significant effect on improving BMI. The following sections will explore the impact of *Moringa oleifera* on these specific indicators in people living with HIV.

The meta-analysis revealed that *Moringa oleifera* supplementation significantly increased CD4^+^ T cell counts in individuals living with HIV (SMD: 1.4, 95% CI: 0.59–2.20, *p* < 0.001), suggesting a beneficial role in modulating immune function. Subgroup analyses demonstrated consistent findings regardless of study design (RCT vs. non-RCT), and meta-regression further confirmed a significant dose-dependent relationship between *Moringa oleifera* intake and increases in CD4+ T cell counts. This effect is likely associated with the abundance of bioactive components in *Moringa oleifera*, including vitamin A, C, and E, zinc, and polyphenols (34–36), which have been reported to promote T cell proliferation, scavenge reactive oxygen species, and reduce cellular apoptosis (37–43).

CD4^+^ T cells are a critical component of the immune system, playing a central role in regulating immune responses and assisting other immune cells such as B cells and cytotoxic T lymphocytes (44, 45). HIV primarily targets these CD4^+^ T cells, leading to their depletion and consequent immunosuppression (46). CD4^+^ T cell count is a key indicator for assessing immune function in HIV-infected individuals and is widely used to monitor disease progression and treatment efficacy (47, 48). When CD4+ T cell counts fall below 200 cells/μl, patients are at high risk for severe opportunistic infections. Therefore, maintaining CD4^+^ T cell numbers and function is vital for the health and prognosis of individuals living with HIV (47, 49). The immunomodulatory effects of *Moringa oleifera* on CD4^+^ T cell counts may involve several possible biological mechanisms. First, its rich antioxidant content can neutralize free radicals, mitigating oxidative stress-induced damage to immune cells (50–52). Oxidative stress is a major contributor to CD4^+^ T cell apoptosis, and its reduction helps preserve CD4^+^ T cell viability and function (53–55). Second, active compounds in *Moringa oleifera*, such as polyphenols and flavonoids, may enhance CD4^+^ T cell proliferation by modulating intracellular signaling pathways. For example, some studies have shown that *Moringa oleifera* extracts activate the PI3K/Akt pathway (56), which is essential for cell growth and survival, thereby promoting CD4^+^ T cell expansion (57, 58). Additionally, *Moringa oleifera* may enhance T cell activity and function by modulating cytokine secretion, further contributing to increased CD4^+^ T cell counts (59). Finally, its immunomodulatory properties may also be linked to its effects on the gut microbiota. A healthy gut microbiome is critical for immune homeostasis, and *Moringa oleifera* may support immune function indirectly by improving microbial composition in the gut (60–64).

Regarding WBC count, our analysis revealed that *Moringa oleifera* significantly increased leukocyte levels (SMD: 0.22, 95% CI: 0.02–0.42), with minimal heterogeneity. This effect may be attributed to the antioxidant properties of *Moringa oleifera*, its capacity to improve nutritional status, and its regulatory effects on hematopoiesis in the bone marrow. Studies have also indicated that folate, iron, and protein contained in *Moringa oleifera* can directly or indirectly promote granulocyte production (65). Changes in WBC count are closely associated with the onset and progression of various diseases. In particular, in individuals living with HIV, a decline in WBC count is often indicative of immune system impairment and increased susceptibility to infections (66). Animal studies have confirmed that *Moringa oleifera* extracts can significantly enhance WBC count and lymphocyte proliferation, suggesting that it may enhance immune responses by promoting the production and activation of leukocytes (67). This immunomodulatory potential is likely related to specific bioactive components in *Moringa oleifera*, such as quercetin and chorogenic acid, which have been shown to influence immune cell function by modulating cytokine secretion (68, 69). Collectively, these findings provide a theoretical foundation for the use of *Moringa oleifera* in individuals with HIV, particularly in improving WB counts and enhancing immune function.

The results of our study on the effects of *Moringa oleifera* on platelet count are also of significant interest. The meta-analysis reveals that *Moringa oleifera* can significantly increase platelet count (SMD: 3.07, 95% CI: 2.77–3.37). It is well-established that platelets are involved not only in hemostasis but also in the release of various cytokines, contributing to both innate and adaptive immune responses (70–72). Therefore, the regulatory effect of *Moringa oleifera* on platelets may enhance their immune recognition and signaling functions, thereby further promoting immune system recovery in HIV patients. The underlying mechanism may be associated with the abundant antioxidant components and bioactive compounds in *Moringa oleifera*, which help alleviate oxidative stress and promote the proliferation and differentiation of hematopoietic stem cells, thus increasing platelet production (73–77). Additionally, specific components of *Moringa oleifera* may influence platelet function and lifespan by modulating cytokine release. For example, *Moringa oleifera* extracts may indirectly promote platelet generation and function by lowering the levels of inflammatory factors (78–80).

BMI is an important indicator reflecting the nutritional status and quality of life of individuals infected with HIV. Our meta-analysis shows that supplementation with *Moringa oleifera* significantly improved BMI in HIV patients (SMD: 0.29, 95% CI: 0.03–0.55). This improvement may hold potential clinical significance in HIV population in developing countries or those with a tendency toward cachexia. *Moringa oleifera* is rich in plant proteins and various vitamins, making it a high-nutritent-density supplement. It may enhance BMI by improving nutritional intake, stimulating appetite, and promoting digestion and absorption (35, 81, 82). Some studies have also reported that *Moringa oleifera* can improve gut microbiota balance, thereby enhancing energy utilization efficiency (60, 83). Moreover, the anti-inflammatory properties of *Moringa oleifera* may help control weight by improving metabolic health and reducing obesity-related inflammatory responses, which could contribute to BMI improvement (84). Moreover, the anti-inflammatory properties of *Moringa oleifera* may help control weight by improving metabolic health and reducing obesity-related inflammatory reactions, contributing to BMI improvement. However, since BMI is a general measure and does not distinguish between changes in fat and muscle mass, future research should incorporate body composition analysis tools (such as Dual-energy X-ray absorptiometry or Bioelectrical impedance analysis) to further evaluate the nutritional intervention effects of *Moringa oleifera* (85).

Emerging evidence suggests that the nutritional and immunomodulatory effects of *Moringa oleifera* may be partly mediated through modulation of the gut microbiota (86). Dysbiosis is a well-documented feature of chronic HIV infection, characterized by reduced microbial diversity, depletion of beneficial commensals such as *Bifidobacterium* and *Lactobacillus*, and expansion of potentially pathogenic taxa (87–89). These alterations contribute to microbial translocation, chronic systemic inflammation, impaired mucosal immunity, and suboptimal nutritional status (90–96). Preclinical studies and limited human trials indicate that *Moringa oleifera* leaves—rich in polyphenols, dietary fibers, and bioactive peptides—can selectively promote beneficial bacteria and suppress harmful species (97–99). Polyphenols can act as substrates for microbial fermentation, leading to increased short-chain fatty acid production, especially butyrate, which supports epithelial integrity, reduces gut permeability, and attenuates microbial translocation, a major driver of immune activation in HIV (100–104). Enhanced gut barrier function and restored microbial composition may in turn improve nutrient absorption and metabolic efficiency, contributing to BMI improvement (105–108). Although direct evidence in HIV populations is scarce, these mechanistic pathways underscore the potential of *Moringa oleifera* to restore gut micro-ecological balance, thereby synergistically supporting immune reconstitution and nutritional recovery. Future randomized controlled trials incorporating microbiome and metabolome profiling are warranted to confirm these effects.

The subgroup analysis and meta-regression analysis revealed the influence of different study designs and intervention dosages on the outcomes. Firstly, supplementation with *Moringa oleifera* significantly increased CD4^+^ T cell counts regardless of whether the study design was a randomized controlled trial or not, suggesting that the intervention effect of *Moringa oleifera* is consistent across different study designs. Secondly, the meta-regression analysis further identified a dose-dependent relationship between the dosage of *Moringa oleifera* and the immune improvement effect. Therefore, further studies should focus on optimizing the dosage and intervention protocols of *Moringa oleifera* to achieve the best therapeutic effect. Additionally, although there were considerable differences in treatment duration across the studies, our regression analysis did not find an impact of treatment duration on efficacy. This lack of association may indicate that the mechanism of action of *Moringa oleifera* is rapidly activated rather than depending on long-term accumulation. Extending the treatment duration from 2 to 12 months may not significantly improve the effect beyond a certain point. The dosage of *Moringa oleifera* had a more significant impact on efficacy (*p* = 0.007), suggesting that the dosage may be more critical than treatment duration in determining the therapeutic effect. Therefore, further research should focus on optimizing the dosage of *Moringa oleifera* rather than solely extending the treatment duration.

Although existing literature suggests that short-term use of *Moringa oleifera* does not appear to have significant adverse effects on kidney or liver function, and no other notable side effects have been reported, we believe that more evidence is needed to support the safety of long-term use of *Moringa oleifera*. Current studies have not found any evidence of toxicity related to *Moringa oleifera* use, suggesting that it may have good safety profiles at standard doses; however, due to the lack of large-scale, long-term safety of *Moringa oleifera* at various doses and in different populations, particularly in groups with compromised immune function, such as individuals living HIV—its safety cannot yet be confirmed.

This pooled analysis has several significant advantages. Firstly, it is the first meta-analysis to evaluate the impact of *Moringa oleifera* intervention on immune function and nutritional status in individuals with HIV, specifically focusing on sub-Saharan Africa, a region with a high HIV burden. This focus holds significant practical implications and population relevance, providing robust support for the evidence-based application of traditional plant therapies in this area. Secondly, the studies included in this meta-analysis span a recent period, from 2016 to 2024, and cover a broad geographic area, including several African countries such as Nigeria, South Africa, Uganda, the Congo, and Kenya, thereby enhancing the representativeness and generalizability of the findings. Additionally, the total sample size of 1,022 participants is significantly larger than that of previous individual, scattered studies, improving the statistical power of the conclusions. Methodologically, this study includes RCTs and non-RCT clinical studies, achieving a good balance between internal and external validity. We systematically assessed the efficacy of *Moringa oleifera* intervention in multiple clinical indicators, including CD4^+^ T cell counts, WBC counts, platelet levels, and BMI, with a broad range of indicators that cover both immune function and nutritional status. The results indicate that *Moringa oleifera* has multiple potential benefits that hold crucial clinical reference values. Furthermore, we conducted various supplementary analyses to improve the reliability of the results. Subgroup analyses revealed that study design type influences heterogeneity to some extent, and meta-regression further identified a dose-response relationship between *Moringa oleifera* dosage and immune improvement, suggesting that intervention intensity may be a key factor influencing efficacy. This finding has practical implications for optimizing intervention dosage in future studies. Publication bias assessment did not identify significant effects from any individual study on the overall results, nor did we observe substantial publication bias, which strengthens the robustness and credibility of the study's conclusions. Finally, the study strictly adhered to the PRISMA and Cochrane guidelines for literature screening, data extraction, and bias assessment, ensuring the scientific rigor and transparency of the research process and providing a reliable methodological foundation for future related studies.

However, this study also has certain limitations. First, all the included studies were conducted in African countries, which may affect the representativeness of the analysis results. However, these studies focus on regions in Africa with high HIV prevalence, and this geographic concentration reflects the widespread use of *Moringa oleifera* in traditional African medicine. This geographic focus has strong practical relevance and reference value, providing preliminary and systematic evidence of the effectiveness of *Moringa oleifera* interventions in this region. Therefore, assessing the therapeutic potential of *Moringa oleifera* in high HIV-burden areas remains important. Second, this study primarily focused on immune indicators (such as CD4^+^ T cells, WBC, and platelets) and only one nutritional indicator (BMI), but it did not include more comprehensive outcome measures such as quality of life and viral load due to the limited availability of relevant data in the current literature. Nonetheless, these core indicators, as early response variables for the potential efficacy of *Moringa oleifera* in HIV patients, still carry significant clinical value. Other important indicators should be explored in future meta-analyses as more studies become available. Finally, the longest intervention duration included in this study was 12 months, and there is a lack of long-term follow-up data to assess the sustained efficacy of *Moringa oleifera* in HIV management. However, the timeframe covered in this study is sufficient to demonstrate its short-term effects on immune function and nutritional status, providing a foundation for future long-term studies.

## 5 Conclusions

This study found that supplementation with *Moringa oleifera* may have a positive effect on increasing CD4^+^ T cell count, as well as leukocyte and platelet levels, while also improving BMI. These findings suggest that *Moringa oleifera* may have the potential for multi-dimensional modulation of immune and nutritional metabolism. However, the above conclusion should be interpreted with caution. Future research should involve more rigorously designed RCTs with larger sample sizes and more standardized intervention protocols to further clarify the mechanisms and clinical effects of *Moringa oleifera*. The significance of this study lies in providing a potentially low-cost, accessible nutritional and immune support supplement for HIV-infected populations in resource-limited regions. Future research should focus on the impact of *Moringa oleifera* on broader outcomes such as disease progression, viral load control, and quality of life, as well as explore its feasibility and safety in combination with existing antiretroviral treatments. Additionally, some *in vitro* and animal studies have suggested that *Moringa oleifera* may have antiviral potential, which is a direction worth further investigation (20, 30, 109).

## Data Availability

The original contributions presented in the study are included in the article/supplementary material, further inquiries can be directed to the corresponding author.
